# Whole-genome resequencing of wild and cultivated cannabis reveals the genetic structure and adaptive selection of important traits

**DOI:** 10.1186/s12870-022-03744-0

**Published:** 2022-07-27

**Authors:** Xuan Chen, Hong-Yan Guo, Qing-Ying Zhang, Lu Wang, Rong Guo, Yi-Xun Zhan, Pin Lv, Yan-Ping Xu, Meng-Bi Guo, Yuan Zhang, Kun Zhang, Yan-Hu Liu, Ming Yang

**Affiliations:** 1 Industrial Crops Research Institute, Yunnan Academy of Agricultural Sciences, Kunming, 650205 China; 2 State Key Laboratory for Conservation, School of Life Sciences, Utilization of Bio-Resources in Yunnan, Yunnan University, Kunming, 650500 China; 3 State Key Laboratory of Genetic Resources and Evolution, Yunnan Laboratory of Molecular Biology of Domestic Animals, Kunming Institute of Zoology, Chinese Academy of Sciences, Kunming, 650223 China

**Keywords:** Cannabis, Wild, Cultivated, Whole-genome resequencing, Genetic structure, Flowering

## Abstract

**Background:**

Cannabis is an important industrial crop species whose fibre, seeds, flowers and leaves are widely used by humans. The study of cannabinoids extracted from plants has been popular research topic in recent years. China is one of the origins of cannabis and one of the few countries with wild cannabis plants. However, the genetic structure of Chinese cannabis and the degree of adaptive selection remain unclear.

**Results:**

The main morphological characteristics of wild cannabis in China were assessed. Based on whole-genome resequencing SNPs, Chinese cannabis could be divided into five groups in terms of geographical source and ecotype: wild accessions growing in the northwestern region; wild accessions growing in the northeastern region; cultivated accessions grown for fibre in the northeastern region; cultivated accessions grown for seed in northwestern region, and cultivated accessions in southwestern region. We further identified genes related to flowering time, seed germination, seed size, embryogenesis, growth, and stress responses selected during the process of cannabis domestication. The expression of flowering-related genes under long-day (LD) and short-day (SD) conditions showed that Chinese cultivated cannabis is adapted to different photoperiods through the regulation of *Flowering locus T-like* (*FT-like*) expression.

**Conclusion:**

This study clarifies the genetic structure of Chinese cannabis and offers valuable genomic resources for cannabis breeding.

**Supplementary Information:**

The online version contains supplementary material available at 10.1186/s12870-022-03744-0.

## Introduction

Cannabis (*Cannabis sativa* L.) is regarded as one of the oldest crop species in the world [1]. This plant is economically important because of its multiple uses. For example, its bast fibre is used for cordage, paper or textiles; its seeds are used for nutrition-related purposes; its flower clusters are used for medicinal or psychoactive drugs; and other parts of the plants are used for various applications, such as cosmetics, personal care products and construction materials. Cannabis produces more than 100 cannabinoids [2, 3], which mainly include tetrahydrocannabinol (THC), cannabidiol (CBD) and cannabigerol (CBG). These three unique compounds have been thoroughly studied and demonstrated to have great potential in the treatment of diseases such as multiple sclerosis, Alzheimer’s disease, epilepsy, depressive disorder and cancer and for the alleviation of pain [4]. In recent years, cannabis has received much attention, and its potential are increasingly positive with the trend for global legalization of medical cannabis and industrial hemp in many countries.

Cannabis is a dioecious annual plant species belonging to the *Cannabis* genus in the Cannabaceae family. The specific epithet has not been decided among members of the academic community. Some botanists [5–9] accept an interpretation with two (or three) species (*C. sativa*, *C. indica* and *C. ruderalis*). However, many scientists propose only a single species of cannabis (*C. sativa*) but with the inclusion of two or three subspecies (subsp. *sativa*, subsp. *indica* and subsp. *ruderalis*) because of the absence of evidence for reproductive barriers to interbreeding among these *Cannabis* populations [10–14].

Cannabis is widely regarded as indigenous to Eurasia [8]. The plants grow during the warm season and need well-drained soils, rich nutrient supplies and sufficient amounts of sunlight [8, 15]. To date, the exact origin of cannabis before human cultivation has not been identified. Either Central Asia or China is most frequently cited as the origin of cannabis domestication [16, 17]. Central Asia, possibly Tajikistan, Afghanistan, Kyrgyzstan and the Xinjiang Region of China, has been identified as the centre of biodiversity for cannabis based on field observations and may be the original centre of domestication [18]. Cannabis cultivation in China for textiles (fibres) or food (seeds) can be traced back at least 6,000 years, and the use of cannabis for medicinal or mystical attributes can be traced back 2700 years, based on archaeological evidence and ancient literature [1, 19].

As one of the first countries to use cannabis, China has become a major country of cannabis cultivation, accounting for approximately 50% of the global cultivation area [20]. China also has an abundance of cannabis germplasm resources across most of its mainland, ranging from approximately 23°N to 51°N, excluding the southeastern coastal areas [14]. Most Chinese resources are landraces and have been domesticated for hundreds of years for different purposes, which have gradually evolved into different local types, such as seed types, fibre types, medicinal types and other local types. Taxonomists recognize different cannabis population types based on their natural origins, agronomic characteristics, and associations with humans [8, 12]. It is surprising that there are still many wild-like cannabis populations growing spontaneously in some areas, mainly distributed in the northeastern, northwestern and southwestern regions of China [21]. Compared with domesticated populations, wild populations generally grow in barren fields without human disturbance and usually show characteristics such as small seed size and easy seed shattering [11, 21, 22]. The abundance of cannabis resources in China, especially wild plants, provides an excellent opportunity to investigate the genetic structure and domestication of cannabis.

Various molecular markers have been used to study the genetic diversity of cannabis. Using chloroplast DNA, Zhang et al. [14] divided Chinese cannabis into three haplogroups that exhibited high, middle, and low latitudinal distribution patterns. However, the wild population could not be distinguished from the domesticated population. Zhang et al. [23] analysed the genetic diversity and population structure of 199 germplasm resources from 12 countries (China, Germany, Hungary, Poland, Ukraine, Lithuania, etc.) by the use of genomic simple sequence repeat (SSR) markers and showed that germplasm resources from different regions were clustered into the same class and that only two subgroups were apparent. With the publication of the first cannabis genome in 2011 [24], the genomic data of 13 different cannabis accessions have been added to the NCBI database, laying a foundation for whole-genome resequencing and high-throughput genotyping of cannabis. By performing global, large-scale, whole-genome resequencing, Ren et al. [25] revealed the domestication history of cannabis, which showed that cannabis was first domesticated in early Neolithic times in East Asia and that current hemp and drug cultivars worldwide diverged from an ancestral gene pool represented by wild plants and landraces in China. Moreover, there have been few studies on wild cannabis outside China, and most of them are focused on the genetic diversity or population structure of marijuana and hemp [26, 27]. In short, there has been no systematic research on wild cannabis in China, and the genetic structure of Chinese cannabis is still poorly understood.

In the present study, we first collected rare wild resources and representative cultivated cannabis resources in China (a total of 21 accessions) and identified their typical agronomic traits. Genomic data of 21 cannabis accessions were obtained through whole-genome resequencing. Through combination of 26 published Chinese cannabis and 5 representative foreign cannabis genomic data, the genetic diversity and population structure of Chinese cannabis were analysed, and the genes related to important traits during the domestication process of cultivated cannabis were further identified. Finally, we focused on flowering time and studied the expression of flowering time-related genes. Our results will improve the understanding of the genetic structure and selections associated with domestication of cannabis in China and provide valuable genomic resources for cannabis breeding.

## Results

### Morphological and physiological characterization

**Fig. 1:**
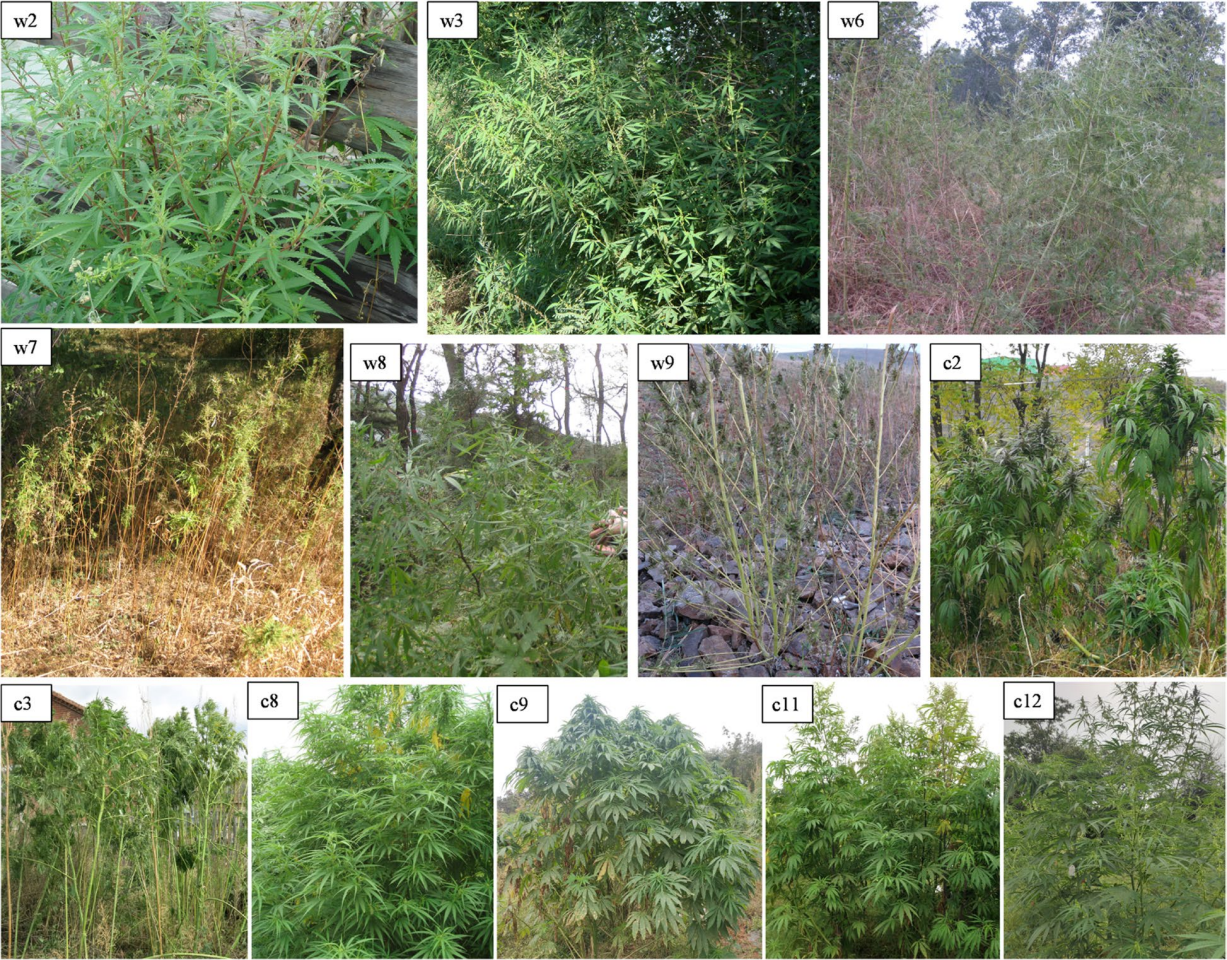
Appearances of representative wild and cultivated cannabis accessions in their original growing areas. Sample identification: w, wild cannabis; c, cultivated cannabis.''

**Fig. 2:**
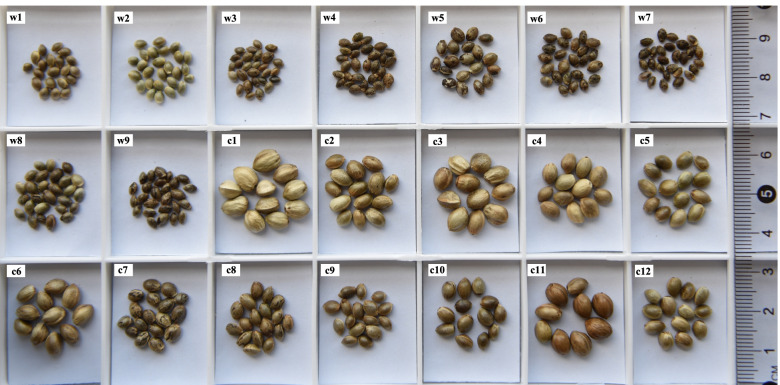
Morphology of seeds of 21 accessions collected across China. Sample identification: w, wild cannabis; c, cultivated cannabis

**Fig. 3:**
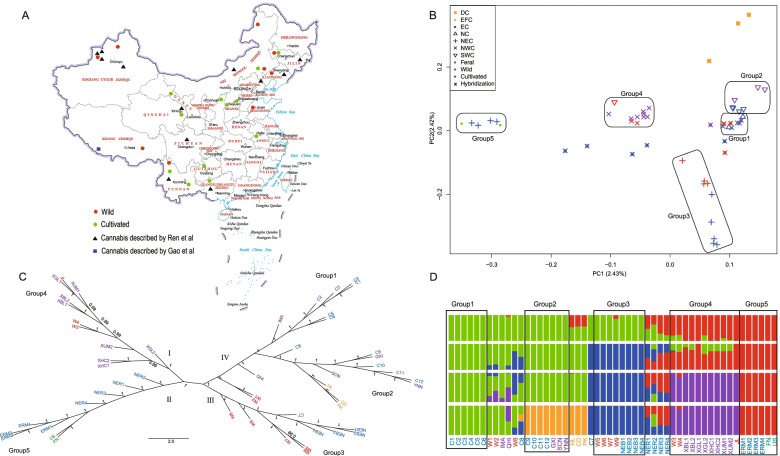
Geographic distribution and population structure of different cannabis accessions. **A** Geographic locations of the Chinese accessions. The map was downloaded from the website of the Ministry of Natural Resources of the People's Republic of China (http://bzdt.ch.mnr.gov.cn), and the drawing review number is GS (2019) 1659. Each red or green dot on the map represents one accession, and these samples were collected by the author. The black triangle and blue box indicate the sample locations described by Ren et al. [25] and Gao et al. [28], respectively. **B** Principal component analysis results of 52 samples (including five samples outside China). DC: Drug cannabis (marijuana), EFC: European cannabis grown for fibre, EC: Eastern China Cannabis, NC: Northwestern China cannabis, NEC: Northeastern China cannabis, NWC: Northwestern China cannabis, SWC: Southwestern China Cannabis. **C** Neighbour-joining tree of the 52 samples based on all the SNPs identified, with 1000 bootstrap replications. The values at the nodes represent the bootstrap values. **D** Population structure of the 52 samples. Each colour represents one population. Each sample is represented by a vertical bar, and the length of each coloured segment represents the proportion contributed by ancestral populations

We collected 21 accessions from 14 provinces in China (Table 1, Fig. 3A). Among these accessions, nine were considered to be wild cannabis, while 12 were cultivated cannabis, which included 10 landraces and two breeding varieties, based on experience and phenotypic characteristics observed in their original growing areas (Fig. 1). To confirm that differences among the accessions were mainly caused by the environment or genetics, we planted both the wild and cultivated cannabis as part of field experiments in Kunming. Obvious differences were observed (Table S1 and Fig. 2); hence, genetic differences caused the phenotypic differences between the wild and the cultivated cannabis. One important difference was that wild cannabis produced small seeds (ranging from 3.15 to 9.80 g, with a mean of 6.86 g/1000 grains) compared with the larger seeds of cultivated cannabis (ranging from 17.40 to 63.13 g, with a mean of 34.24 g/1000 grains). The two sets of data did not overlap, so they are obviously different (Fig. S1). Mature seeds from wild plants fell off the pedicel easily, and most wild seeds had an obvious fleshy caruncle at the base (an elongated attachment base). Germination tests showed that the natural germination rate of wild seeds was less than 2% at room temperature, and cold (4 °C) and wet stratification treatments were necessary for germination of the wild seeds (Table S1).

**Table 1:** Sample information

Accession name	Sample ID	Location/origin	Latitude(°N)	Type	Seed weight (g/1000 grains)	Camouflage covering (Yes or No)
W163	W1	Yunnan, SW China	27.70	W	5.63	N
W270	W2	Xizang, SW China	29.59	W	5.42	N
W606	W3	Xinjiang, NW China	43.92	W	3.15	Y
W274-C	W4	Xinjiang, NW China	43.48	W	6.91	Y
W254-B	W5	Inner Mongolia, NE China	41.50	W	9.80	Y
W594	W6	Liaoning, NE China	42.68	W	9.56	Y
W596	W7	Jilin, NE China	45.06	W	5.78	Y
W50	W8	Shandong, E China	36.41	W	8.29	Y
W645-A	W9	Inner Mongolia, NE China	50.16	W	7.19	Y
C466	C1	Qinghai, NW China	36.50	L	48.34	N
C294	C2	Gansu, NW China	39.42	L	33.61	N
C263	C3	Inner Mongolia, NW China	42.15	L	52.45	N
C480	C4	Shaanxi, NW China	38.28	L	38.32	N
C623	C5	Shanxi, N China	37.87	B	29.22	N
C602	C6	Shanxi, N China	37.43	L	40.96	N
C597	C7	Jilin, NE China	45.06	L	19.32	Y
Lu'an HanMa	C8	Anhui, E China	31.45	L	18.53	Y
Bama HuoMa	C9	Guangxi, SW China	24.15	L	17.40	N
C197	C10	Guizhou, SW China	26.66	L	25.36	N
C102-B	C11	Yunnan, SW China	24.24	L	63.13	N
YunMa1	C12	Yunnan, SW China	26.11	B	24.27	N
-	JL	Xizang, SW China [28]	28.47–29.10	W	-	-
-	XHC1	NW China [25]	44.25	F	-	-
-	XHC2	NW China [25]	44.25	F	-	-
-	XGL1	NW China [25]	43.49	F	-	-
-	XGL2	NW China [25]	43.49	F	-	-
-	XBL1	NW China [25]	44.92	F	-	-
-	XBL2	NW China [25]	44.92	F	-	-
-	XUM1	NW China [25]	43.77	F	-	-
-	XUM2	NW China [25]	43.77	F	-	-
R1in136	ERM1	NE China [25]	45.51	B	-	-
R1in136	ERM2	NE China [25]	-	B	-	-
R1in136	ERM3	NE China [25]	-	B	-	-
R1in136	ERM4	NE China [25]	-	B	-	-
R2in135	NER1	NE China [25]	44.17	L	-	-
R2in135	NER2	NE China [25]	-	L	-	-
R2in135	NER3	NE China [25]	-	L	-	-
R2in135	NER4	NE China [25]	-	L	-	-
R3in134	NEB1	NE China [25]	43.37	L	-	-
R3in134	NEB2	NE China [25]	-	L	-	-
R3in134	NEB3	NE China [25]	-	L	-	-
R3in134	NEB4	NE China [25]	-	L	-	-
-	IMA	N China [25]	43.18	F	-	-
-	QHI	NW China [25]	36.86	F	-	-
-	SCN	SW China [25]	31.35	F	-	-
-	YNN	SW China [25]	24.21	F	-	-
-	GXI	SW China [25]	23.6	F	-	-
Purple Kush	PK	USA (https://www.ncbi.nlm.nih.gov/sra/?term=SRP008673)	-	B	-	-
Chemdawg	CD	https://www.medicinalgenomics.com.	-	B	-	-
Harlequin (14569)	HL	USA (https://www.ncbi.nlm.nih. gov/sra/?term = SRR4446095)	-	B	-	-
Finola	FN	Finland (https://www.ncbi.nlm.nih.gov/sra/?term=SRP008673)	61.98	B	-	-
USO-31	US	Ukraine (https://www.ncbi.nlm.nih.gov/sra/?term=SRP008673)	50.08	B	-	-

*W* Wild (the plants were collected from barren fields, and their phenotypic characteristics were judged), *F* Feral (the plants were collected in the field [25]), *L* Landrace (domesticated, locally adapted, traditional variety), *B* Breeding variety (a cultivar selected by humans for desirable traits), "-" indicates missing data

Except for seeds from Yunnan (W1) and Xizang (W2), the seeds from the other seven wild accessions all had a camouflage covering (a thin dark brown film attached to the surface of a seed), while only two accessions from Jilin (C7) and Anhui (C8) had a small amount of camouflage covering (Fig. 2). Moreover, wild cannabis bloomed earlier than domesticated cannabis. Although the flowering time of W1 and W2 was approximately 55 days, the flowering time of other wild cannabis accessions was shorter than 35 days (Table S1). In addition, the values of the first branch height, petiole length, compound leaf width and leaflet width of wild cannabis were significantly lower than those of cultivated cannabis (Fig. S1). We also observed that, when planted at low latitudes (Kunming), cultivated cannabis (C1-C7) from relatively high latitudes exhibited early flowering, early maturity, a dwarf stature and almost no branches (Fig. S1). However, wild cannabis plants still produced a relatively large number of branches in Kunming.

### Sequencing, variation and diversity

To identify the genetic basis of wild and cultivated cannabis, we used the Illumina HiSeq 2000 platform to perform whole-genome resequencing for the 21 Chinese accessions (Table S2). The sequencing results revealed an average 10.83 × genome coverage depth. Furthermore, genome sequencing data of 25 Chinese cannabis [25], one Chinese wild cannabis [28], three marijuana and two European cannabis accessions grown for fibre were collected from public databases (Table 1). After stringent quality filtering, the high-quality reads were mapped back to the most contiguous and complete chromosome-level assembly of cannabis (cs10/CBDRx, GenBank accession No. GCA_900626175.2) [29, 30]. We identified 22.98 million single-nucleotide polymorphisms (SNPs) located within the nine autosomes and the X chromosome for further analysis. Most of the SNPs (85.00%) were located in intergenic regions, and only 4.93% were located in coding sequence regions (Table S3).

Genetic diversity (θ) (4 Nμ) (Table S4) was obtained for each individual by comparing the two haploid genomes within each individual [31]. Among the 47 cannabis accessions from China, the genetic diversity of cannabis in NE China was quite different. NER had the highest genetic diversity, with an average of 4.63 × 10^–3^, while ERM had the lowest genetic diversity, with an average of 3.80 × 10^–3^. According to the analysis of population structure (Fig. 3D), the average genetic diversities of wild (or feral) cannabis in NE China (Group 3) and in NW China (Group 4) and of cultivated cannabis in NW China (Group 1) were 4.36 × 10^–3^, 4.12 × 10^–3^ and 4.21 × 10^–3^_,_ respectively, while the average genetic diversity of cultivated cannabis in SW China (Group 2) was 4.00 × 10^–3^ (Fig. S2). These results show that cannabis accessions at high latitudes have higher genetic diversity than those at low latitudes do. For cultivated cannabis, we also found that their genetic diversity was significantly positively correlated with latitude (*p* < 0.01) (Fig. S2).

### Population structure of wild and cultivated cannabis

To explore the genetic relationships among cannabis resources, we used a block relaxation algorithm and performed a structure analysis to cluster individuals into different numbers of ancestors (Fig. 3D) [32]. For *K* = 2, we found that northwestern Chinese wild (or feral) cannabis clustered together with European cannabis grown for fibre but was separate from other Chinese cannabis. NERs from NE China were genetic admixture with European cannabis grown for fibre. For *K* = 3, wild (or feral) cannabis in NE China was isolated and constituted an independent subgroup. For *K* = 4, wild (or feral) cannabis in Xinjiang and Xizang further constituted an independent subgroup. When *K* = 5, the cultivated cannabis in SW and NW China were separated from each other and constituted independent subgroups. In addition, three marijuana and cultivated cannabis in SW China clustered into the same subgroup. When *K* = 3, 4 and 5, we found that W1, W2, W8, C8, IMA, QHI and NERs were heterozygotes of two or more groups of genes.

Next, we conducted a principal component analysis (PCA) [33] and constructed a neighbour-joining (NJ) tree [34] comprising the 22.98 million high-quality SNPs. According to the PCA results, all the samples could be divided using the first and second eigenvectors into five groups: 1) cultivated cannabis grown for seed from NW China; 2) cultivated cannabis from SW China; 3) wild (or feral) cannabis from NE China; 4) wild (or feral) cannabis from Xinjiang and Xizang; and 5) European cannabis grown for fibre plus ERMs (Fig. 3B). The NJ tree of Chinese cannabis agreed with the PCA results and population structure. All the samples could be divided into four clades according to geographic clustering (Fig. 3C), including clade I with wild (or feral) cannabis in Xinjiang and Xizang, clade II with European cannabis grown for fibre together with ERMs and NERs from NE China, clade III with wild (or feral) cannabis collected from NE China, and clade IV with cultivated cannabis in NW and SW China. Moreover, W1 and W2 from SW China were clustered between clades III and IV. The three marijuana genotypes were genetically most similar to the cannabis accessions from SW China.

### Genes selected during domestication

**Fig. 4:**
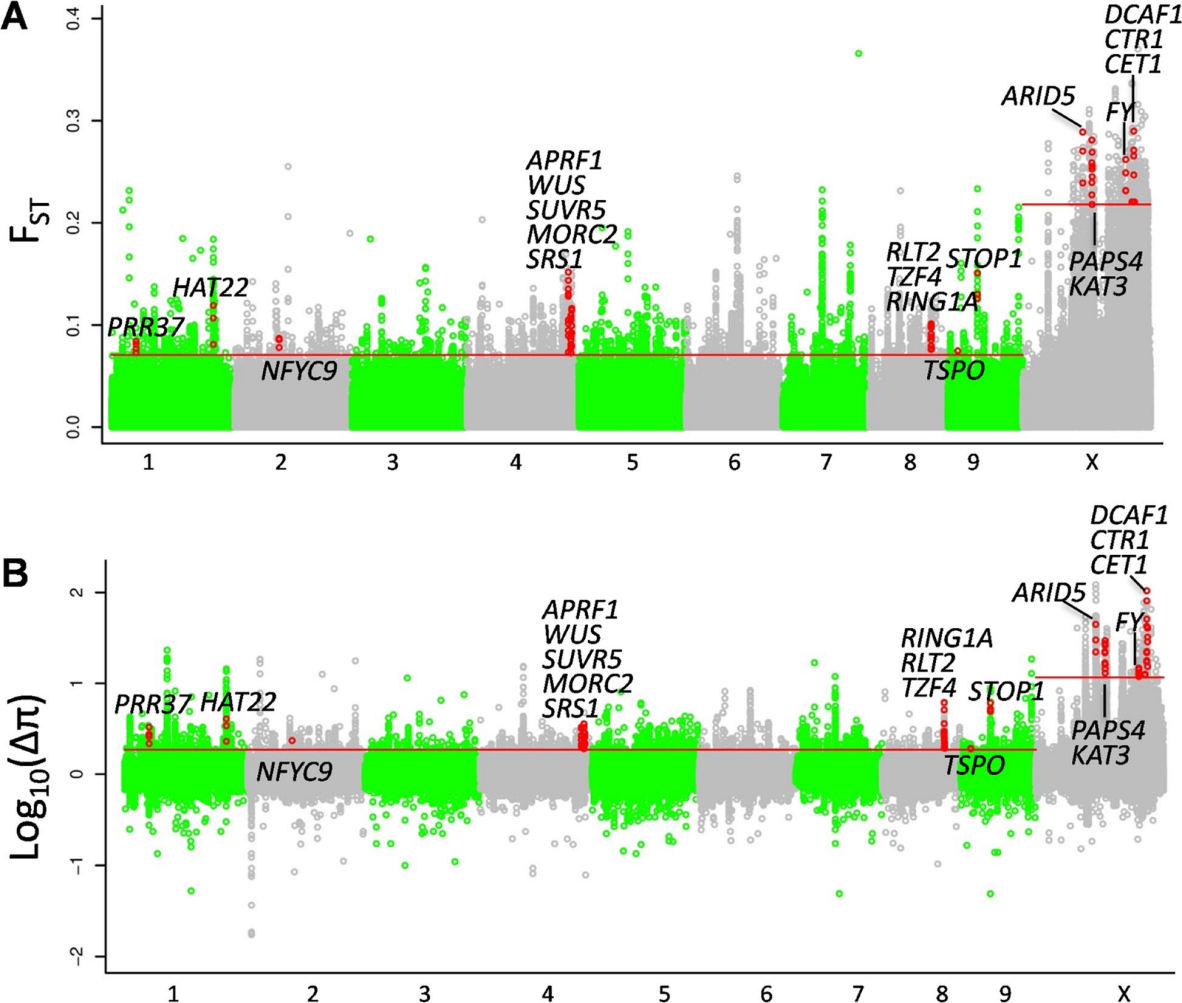
Selection scans of cultivated cannabis. **A** Genomic landscape of FST values between cultivated cannabis and wild cannabis. **B** Genomic landscape of Δπ values (θπ_wild_/θπ_cultivated_). The red lines indicate the top 1% value. The red dots indicate the values of the genes at the corresponding positions

We observed and recorded several different phenotypes among Chinese wild and cultivated cannabis. To explore their genetic basis, we used the coefficient of nucleotide differentiation (F_ST_) and the difference in nucleotide diversity across populations (Δ_π_) to identify positively selected signals after outliers and admixed individuals were excluded. The X chromosome is more sensitive to domestication history and selective effects than autosomes are [35, 36]. For each method, the top 1% windows of autosomes and the X chromosome were separately selected for gene annotation. Overall, we identified 209 common positive selection genes (PSGs) according to F_ST_ (804 PSGs) and Δ_π_ (598 PSGs) values (Table S5, Fig. 4).

Among the 209 common PSGs, nine are related to flowering. CENTRORADIALIS (CEN)-like protein 1 (encoded by *CET1*) is strongly expressed in developing inflorescences in *Arabidopsis* and *Antirrhinum* [37, 38]. Overexpression of this gene delays flowering and alters flower architecture in *Hevea brasiliensis* [39]. Histone-lysine N-methyltransferase (SUVR5) mediates H3K9me2 deposition and affects flowering time by binding Lysine-specific Histone Demethylase 1 homologue 1 (LDL1) [40]. FY is an RNA 3' end-processing factor that interacts with FCA to regulate flowering time [41]. The putative PRC1 RING-finger protein (RING1A) regulates the vegetative phase transition by affecting the expression of the *Squamosa Promoter Binding Protein-like* (*SPL*) gene [42]. Loss of function of *AtRING1A* results in a late-flowering phenotype by repressing MADS Affecting Flowering 4/5 [43]. Nuclear poly(A) Polymerase 4 (PAPS4) creates the 3'-poly (A) tail during maturation of pre-mRNAs, which affects mRNA stability [44]. Overexpression of *PAPS4* results in earlier flowering and reduces *Flowering Locus C* (*FLC*) expression in *Arabidopsis* [45]. Anthesis Promoting Factor 1 (APRF1) acts upstream of *FLC* and promotes flowering under long days in *Arabidopsis thaliana* [46]. Loss of function of *APRF1* was shown to delay flowering, and overexpression of *APRF1* accelerates flowering. Pseudoresponse Regulator protein 37 (PRR37) regulates heading and controls flowering time by negatively regulating the expression of *HD3A* [47]. Nuclear Transcription Factor Y subunit C-9 (*NFYC9*) physically interacts with CONSTANS (CO), a key regulator of photoperiod-dependent flowering time, and is genetically required for CO-mediated floral promotion [48]. The DDT domain-containing protein RINGLET2 (RLT2) has been shown to activate the vegetative-to-reproductive transition that in turns regulates the expression of several key genes to affect flowering time [49].

We also identified seven PSGs related to seed germination and plant development. Zinc finger CCCH domain-containing protein 2 (TZF4), a transcriptional regulator, affects seed germination by controlling the expression of genes critical for ABA and GA responses in *Arabidopsis* [50], and Small and Round Seed 1 (SRS1) regulates rice seed size by reducing both cell length and cell numbers in the longitudinal direction [51]. Translocator proteins (TSPO) modulate storage lipids and cytoplasmic lipid droplet metabolism in seeds of *Arabidopsis* [52]. DDB1-CUL4 Associated Factor homologue 1 (DCAF1) is essential for plant embryogenesis, and reduced levels lead to various developmental defects [53]. The serine/threonine protein kinase Constitutive Triple Response 1 (CTR1) is a negative regulator of the ethylene response pathway in *Arabidopsis* [54]; ethylene is important for plant growth, development and stress responses [55]. AT-rich Interactive Domain-containing protein 5 (ARID5) is a subunit of a plant-specific imitation switch complex and regulates development and floral transition in *Arabidopsis* [56]. WUSCHEL (WUS) plays an important role in regulating stem cell fate throughout development [57], and mutations in this gene result in the failure of the self-maintenance of both shoot and floral meristems [58].

Furthermore, we identified four genes related to stress responses. Sensitive to Proton Rhizotoxicity 1 (STOP1), a zinc finger transcription factor, regulates tolerance to various stresses in *Arabidopsis*. For example, *STOP1* is activated to rapidly inhibit root cell elongation under external phosphate-limiting conditions [59]. STOP1 is also crucial for proton and aluminium tolerance in *Arabidopsis* [60], and this protein reduces the expression of *CBL-interacting protein kinase 23* (*CIPK23*) to regulate potassium (K^+^) homeostasis under salt and drought stress [61]. The homeobox-leucine zipper protein HAT22, which is also named ABIG1, is upregulated in response to drought and abscisic acid treatment in *Arabidopsis* [62]. *HAT22* overexpression reduces the chlorophyll content of seedlings and hastens the onset of leaf senescence in *Arabidopsis* [63]. MICRORCHIDIA 2 (MORC2) contributes to resistance against disease and pathogen-associated molecular immunity triggered by R proteins [64, 65]. The K^+^ channel encoded by *KAT3*, also known as *AtKC1*, is a Shaker-like K^+^ channel that regulates the uptake and allocation of K^+^ in *Arabidopsis* roots under low-K^+^ stress [66].

### Flowering time and flowering-related gene expression

Although we measured the flowering time of cannabis accessions from different latitudes in China under natural short-day (SD) conditions in Kunming (Table S1), it is necessary to study the flowering response of different cannabis accessions under long-day (LD) conditions. We found that wild cannabis displayed flower buds within 50 days under LD conditions; at only 31 days after planting, flower buds appeared on W9. However, the cultivated cannabis accessions from SW China and NW China remained in a vegetative state at 100 days after planting, and no flower buds appeared. To study the regulatory mechanism of flowering time, we selected four accessions (W9, W4, C4 and C10) from different latitudes to study the expression of flowering-related genes under LD and SD conditions. The four accessions were first subjected to LD conditions and then to SD conditions. The expression of four flowering-related PSGs and three flowering pathway integration or regulatory genes (*FT-like* [67], *SOC1* and *FLC-like*) were analysed. On the basis of the expression results of *FT-like* at different time points on the same day (sampling every 3 h) (Fig. S3) and on previous research results [68], the sampling time was set at 10:00.

**Fig. 5:**
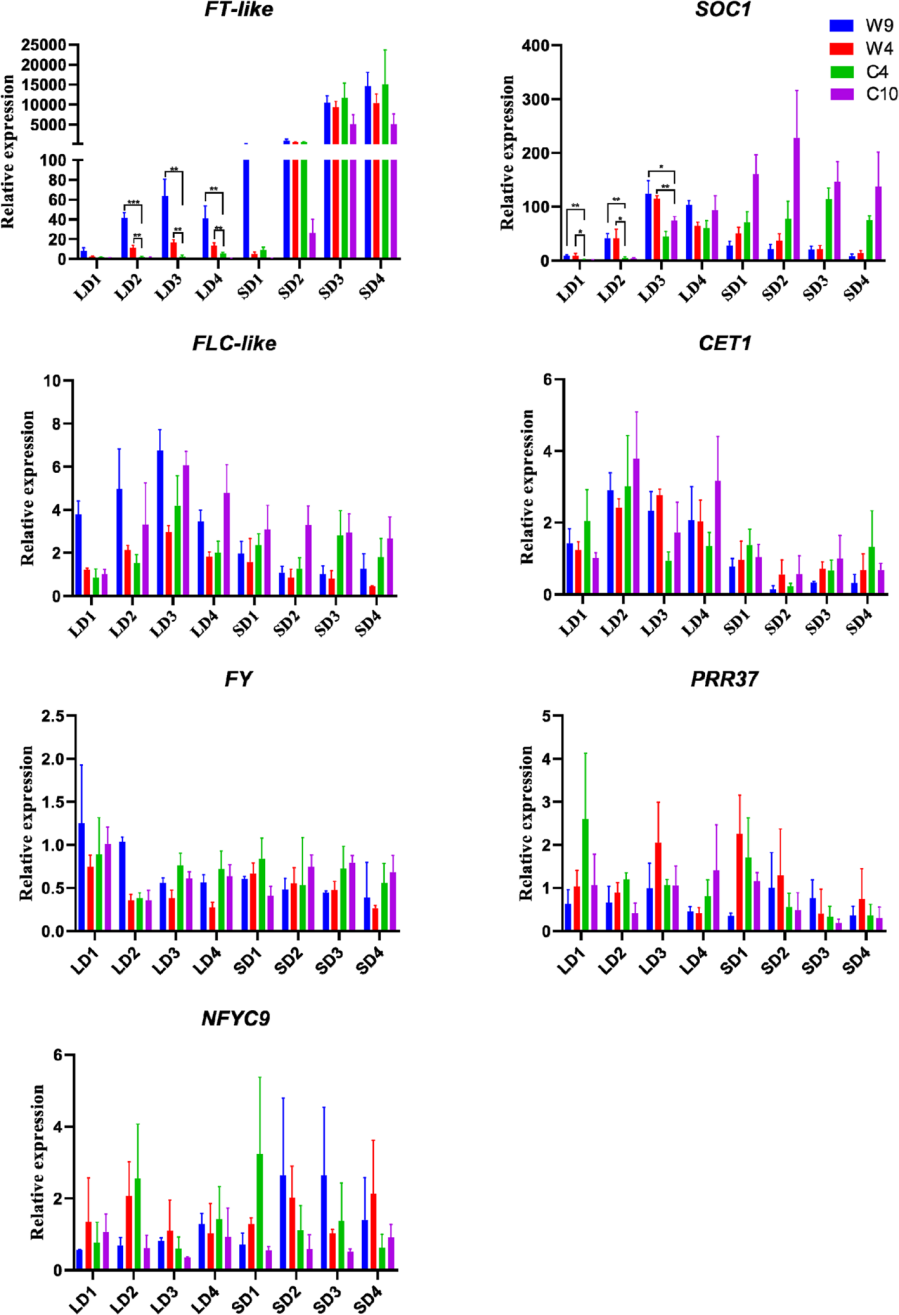
Expression of flowering-related genes in cannabis under LD and SD conditions. LD1-LD4 represent four samples collected under long-day conditions, and SD1-SD4 represent four samples collected under short-day conditions. The data represent the means ± SDs. Significant differences were determined using GraphPad Prism 8 software (* indicates *P* < 0.05; ** indicates *P* < 0.01; *** indicates *P* < 0.001)

W9 and W4 showed flower buds at the timepoints of LD3 and LD4, respectively, while C4 and C10 did not display flower buds until SD3. Under LD conditions, the expression levels of *FT-like* in W9 and W4 were significantly (*p* < 0.01) higher than those in C4 and C10, and the expression of *FT-like* showed a positive correlation with the latitude of the material’s original location (Fig. 5). Under SD conditions, *FT-like* expression was rapidly induced to a high level in all four accessions, with relative expression levels of 10,339, 9228, 11,627 and 4959 at SD3, respectively. Under LD conditions, the expression of *SOC1* in W9 and W4 was also significantly higher than that in cultivated cannabis (Fig. 5). We further determined the expression of two positively regulated flowering time-related PSGs (*FY* and *NFYC9*) and two negatively regulated flowering time-related PSGs (*CET1* and *PRR37*), but they showed little change in expression in the four accessions at different developmental stages (Fig. 5). *A*s a negative regulator of flowering time in the autonomous and vernalization flowering pathways [69, 70], *FLC-like* also exhibited slight changes in expression in the four accessions at different developmental stages, with the maximum relative expression levels exhibiting a sevenfold increase.

## Discussion

Cannabis has been domesticated by humans for thousands of years. China or East Asia is one of the centres of origin of cannabis [25]. Some key questions remain concerning whether pure wild cannabis still exists in China and concerning the nature of the genetic structure of cannabis in China. To answer these questions, we studied the genetic structure of Chinese cannabis from morphological and phylogenetic perspectives. This study showed that Chinese wild cannabis has characteristics that include early flowering, small seed size, a low natural germination rate, the presence of caruncles on the seeds, easy abscission and strong branching. We further analysed the genetic structure of wild and cultivated cannabis in China and divided Chinese cannabis into five groups according to their geographical source and ecotype. Afterwards, the genes related to important traits selected for during the domestication process of cultivated cannabis were also analysed.

We found that the wild-growing resources collected from Xinjiang, NE China and Xizang had the typical characteristics of wild plants both in the origin location and in the Kunming region. Morphological characteristics, such as small seed size, the presence of caruncles on the seeds, easy abscission, a low natural germination rate, early flowering and strong branching, may also present in pure wild cannabis. When these results were combined with the results obtained by Ren et al. [25], regardless of whether the wild China cannabis accessions are truly wild or not (on the basis of their phenotype), it is at least certain that these wild cannabis genotypes are indeed derived from early cannabis ancestors. We also found that wild cannabis is mainly distributed across high-latitude regions in China. At low latitudes, such as in SW China, the wild cannabis (W1 and W2) accessions are heterozygotes of wild and cultivated cannabis. At the same time, there is more genetic exchange within high-latitude cultivated cannabis due to its proximity to wild cannabis growing areas (Fig. 3B, D). For example, NERs in NE China had the highest heterozygosity. Although C7 and NEBs were considered landraces at the time of collection, they clustered into a group with wild cannabis in terms of their phylogeny and population structure. This is consistent with the results of Ren et al. in which NEBs clustered as a basal cannabis [25].

Cannabis is widely distributed from the southern region to northern region of China (approximately 23°N to 51°N, 80°E to 125°E) [14]. There are both wild-type and cultivated types in China, and the cultivation purposes are diverse. Due to its characteristics of being open pollinated and amenable to outcrossing, cannabis has high genetic variation and a high level of heterozygosity, which limits the effects of molecular markers for genetic diversity research and practical applications [28, 71]. Therefore, using a variety of polymorphic molecular markers to explain the genetic diversity of cannabis in China has always been the focus of research. We previously used sequence variations of five chloroplast DNA regions to investigate the phylogeographic structure of cannabis in China, but we identified only three haplogroups exhibiting high-middle-low latitudinal distribution patterns and did not distinguish between wild-type and cultivated types [14]. Zhang et al. [23] used 59 polymorphic SSRs and three phenotypic markers to evaluate 199 cannabis germplasm resources from 12 countries and divided the germplasms into two subgroups: the first group included cannabis outside China and some cannabis accessions in SW China, and the other group included the remaining Chinese cannabis accessions. The phylogenetic tree in this study revealed that Chinese germplasms were not clustered within a certain group and did not exhibit regional classification. Ren et al. [25] focused on elucidating the evolutionary history of cannabis on a global scale. Based on 22.98 million whole-genome resequencing SNPs, our study clearly showed that Chinese cannabis could be divided into five groups in terms of geographical source and ecotype: a wild cannabis group from NW China; a wild cannabis group from NE China; a cultivated cannabis group grown for fibre from NE China; a cultivated cannabis group grown for seed from NW China, and a cultivated cannabis group from SW China. In addition, there were unclear boundaries between wild accessions and some landraces (such as NEBs in this study) in NE China. The types grown for fibre in NE China and European cannabis grown for fibre were very closely genetically related. Marijuana (also known as Indian cannabis) was genetically similar to cannabis in SW China. The most likely reason is that SW China is geographically adjacent to South Asian countries such as India, and there may have been gene exchange between cannabis in the two regions.

Analysis of the five cannabis groups in China showed that the genetic diversity of three northern cannabis populations at high latitudes was greater than that of the cannabis cultivated in SW China (Table S2). This may be because wild cannabis originated at high latitudes, and there was more genetic exchange between wild accessions and landraces near the origin, resulting in higher genetic diversity of cannabis at high latitudes. However, cannabis in SW China, which is geographically far from the growing area of wild cannabis, has been continuously selected by humans, resulting in a slight reduction in genetic diversity. According to a scatterplot of the genetic diversity of cultivated cannabis (Fig. S2B), it can be seen that the genetic diversity of cultivated cannabis in NW China tends to be closer, while that in SW China tends to be scattered. These results may be related to the different traditions and customs of people in the two regions. People in NW China like to eat cannabis fruits, while people in SW China have a variety of traditions involving cannabis, such as eating cannabis fruits, weaving fibre, and other religious activities, because there are many national minority groups living in the southwestern region of China [72, 73].

Throughout the long history of human production activities, wild cannabis has been selected and domesticated by humans for different traits. For example, people have selected nondormant seeds to improve sowing efficiency, large seeds that do not natural shatter to obtain high seed yields, tall plants with few branches to obtain increased yield of bast fibre, and plants with high contents of cannabinoids to meet religious or medicinal needs. According to our genetic selection analysis, we identified several important genes related to flowering time, seed germination, seed size, embryogenesis, growth and stress responses. These genes are helpful to explain the genetic basis of the difference between cultivated cannabis and wild cannabis.

As a short-day crop species, cannabis is sensitive to the photoperiod, and flowering time is greatly influenced by the daylength of the growing season [74]. Generally, planting low-latitude varieties at high latitudes prolongs the growing period but may increase the risk of loss of immature fibre or seeds due to an earlier frost period. Conversely, planting high-latitude varieties at low latitudes shortens the growing period but severely reduces fibre and seed yield [75]. Surprisingly, our study showed that the critical daylength of wild cannabis is very long, and it can still flower even under 18 h of daylight. Therefore, the important factor in the process of cannabis domestication is flowering time. FT (FT-like) is an important integration factor in the photoperiod-induced flowering pathway, autonomous flowering pathway and vernalization-dependent flowering pathway, and FLC-like plays an important role in the negative regulation of the autonomous flowering and vernalization pathways [76]. Our results showed that even under extreme LD conditions, *FT-like* was still highly expressed in wild cannabis and promoted flowering. However, cultivated cannabis from SW and NW China maintained low *FT-like* expression and vegetative growth and exhibited *FT-like* gene expression and flowering only under SD conditions. Moreover, the low level of *FLC-like* expression also indicated that the flowering behaviour of cannabis may not be controlled by the autonomous flowering or vernalization pathways. These results imply that cultivated cannabis has adapted to different photoperiod conditions through the regulation of *FT-like* expression.

## Conclusion

In summary, this study provides a comprehensive analysis of the genetic structure of wild and cultivated cannabis in China. First, we confirmed that Chinese wild cannabis has the characteristics of early flowering, small seed size, a low natural germination rate, the presence of caruncles on the seeds, easy abscission and strong branching. Second, Chinese wild and cultivated cannabis could be divided into five groups according to geographical source and ecotype. We further identified several important genes related to flowering time, seed germination, seed size, embryogenesis, growth and stress responses, which are helpful to explain the genetic basis of the difference between cultivated cannabis and wild cannabis. Third, Chinese cultivated cannabis has adapted to different natural photoperiod conditions through the regulation of *FT-like* expression. However, the specific mechanism regulating flowering time needs to be further examined.

## Methods

### Plant materials and growth conditions

The seeds and leaves of 21 cannabis accessions were collected from plants composing natural populations in China, and seeds of each accession have been deposited in the seed bank of the Industrial Crops Research Institute, Yunnan Academy of Agricultural Sciences, Kunming, China. Among the accessions, nine wild ones nearly covered the entire distribution range of wild cannabis throughout China (Xinjiang, Xizang, Inner Mongolia, Liaoning, Jilin, Shandong and Yunnan Provinces). Twelve domesticated accessions were representative landraces and breeding cultivars in China. After they germinated in bags containing nutrient-enriched media, all the seeds were transplanted into the soil at an experimental field site under natural SD conditions in Kunming (SW China, 102.62°E/25.11°N, day length < 13 h during the vegetative period), and water and fertilizer management was carried out according to field production practices.

### DNA extraction and sequencing

Genomic DNA was isolated from young leaves of samples using the cetyl-trimethylammonium bromide (CTAB) method, with additional steps to remove any protein and RNA [77]. One to three micrograms of DNA of each individual was sheared into fragments of 200–800 bp using a Covaris system (Covaris, Inc.). The DNA fragments were then sequenced using an Illumina HiSeq 2000 platform. In addition, the genomic data of 31 cannabis accessions, namely, 25 Chinese cannabis accessions, one Chinese wild cannabis accession, three marijuana varieties (Purple Kush, Chemdawg and Harlequin) and two European cannabis varieties grown for fibre (Finola and USO-31), were downloaded from public databases. Relevant information concerning the accessions is shown in Table 1.

### Sequence data preprocessing and variant calling

The raw sequence reads were mapped to the cannabis reference genome (GCA_900626175.2) using BWA-MEM version 0.7.8 [78]. The reads with identical start/end points were filtered using PICARD (version 1.87). SNP calling of the sequence data was performed using mpileup of SAMtools (version 0.1.18) [79]. The following filters were used: 1) a miss ratio of less than 50%; 2) QUAL value of more than 40; and 3) keeping of only biallelic SNPs.

## Genetic diversity and population structure

Population structure analysis was performed using the block relaxation algorithm implemented in ADMIXTURE software (1.3.0) [32]. Principal component analysis was carried out using the smartPCA program of the EIGENSOFT package v5.0.1 [33]. An NJ tree was constructed using MEGA (7.0.20) [34]. The genetic diversity (θ) (4 Nμ) of five groups was assessed with 10 kb windows separately by VCFtools v0.1.12b [31].

### Positive selection

Based on the phenotypes, structure results, NJ tree and PCA results, Group 1 and Group 2 comprised cultivated cannabis, and Group 3 and Group 4 comprised wild cannabis. We scanned for positive selection signals across the genomes of cultivated cannabis. Genetic diversity (θπ) and F_ST_ were calculated with 10 kb windows and 2 kb steps across the genome using VCFtools v0.1.12b [31], and Δ_π_ was calculated as Δ_π_ = θπ_wild_/θπ_cultivated_ on a log_10_ scale. For each method, the top 1% windows for autosomes and X chromosomes were retained for gene annotation separately. Genes overlapping in both gene sets were considered significant candidate genes under positive selection.

### Flowering time observations under LD conditions

Seven wild cannabis accessions (W1, W2, W4, W6, W7, W8 and W9) and six landraces (C1, C3, C4, C8, C10 and C11) were selected to study flowering time under LD conditions. After they germinated, the seeds were transplanted into clay pots, which were moved to an artificial climate chamber (25 ~ 28 °C temperature, 70% humidity) and allowed to grow. The light source consisted of LED bulbs imitating natural light. The photoperiod was set such that it mimicked LD conditions (6:00–24:00, 18 h of light/6 h of darkness). The flowering time was recorded when the flower buds were visible at the top of the male plants.

### qRT–PCR analysis of flowering-related genes under LD and SD conditions

After the flowering times of the cannabis plants were recorded, another set of experiments involving two wild cannabis accessions (W9 and W4) and two landraces (C4 and C10) was designed to study flowering-related gene expression. The four accessions originated from different latitudes, namely, 50.16°N, 43.48°N, 38.28°N and 26.66°N. In the first growing stage, the photoperiod was set such that it mimicked LD conditions (6:00–24:00, 18 h of light/6 h of darkness) until there were flower buds present on the wild cannabis plants. In the second growing stage, the photoperiod was set such that it mimicked SD conditions (8:00–18:0, 10 h of light/14 h of darkness) until the cultivated cannabis plants were flowering. Samples were taken at 10:00 every 10 days throughout the whole growth period. A total of 8 samples for each accession during the different periods were taken: 4 samples (named LD1-LD4) under LD conditions and 4 samples (named SD1-SD4) under SD conditions. The sampling location was the first to second pair of true leaves, from the top down. Three biological replicates were taken each time. After sampling, they were quickly put into liquid nitrogen for freezing and stored at -80 °C. Total RNA was extracted using an RNeasy Plant Mini Kit, cDNA was synthesized using EvoScript Universal cDNA Master Mix, and MonAmp™ TaqMan qPCR Mix was used to carry out qRT–PCR (quantitative reverse-transcription PCR) for flowering-related genes; *EF1α* (*Elongation factor 1-alpha*) served as a reference gene, as previously described [80]. The qRT–PCR assays were conducted for three biological replicates, and each biological replicate involved three technical replications. The primers and probes used were designed according to the coding sequences and are listed in Table S6.

### Statistical analysis

For the gene expression experiments, the data were analysed for three biological replicates, and each biological replicate was analysed for three technical replications. For phenotypic data, the samples from the wild subpopulation and cultivated subpopulation were used to determine differences. Significant differences were determined using GraphPad Prism 8 software (* indicates *P* < 0.05; ** indicates *P* < 0.01; *** indicates *P* < 0.001; **** indicates *P* < 0.0001).

### Definitions

#### Marijuana

Drug types of cannabis used for medicinal purposes or for recreation.

#### Hemp

Nondrug-type of cannabis grown for the production of seeds and fibre.

#### Industrial hemp

Hemp varieties for which the maximum tetrahydrocannabinol (THC) content is < 0.3% dry matter of the flowers and leaves of the plant population.

## Supplementary Information

**Additional file 1:** **Table S1.** Morphological and agronomic characteristics of 21 cannabis accessions in Kunming.

**Additional file 2:** **Table S2.** Sequencing reads and mapping rate of 21 cannabis accessions.

**Additional file 3:** **Table S3.** Distribution of SNPs across the whole genome of 52 cannabis accessions.

**Additional file 4:** **Table S4.** The nucleotide diversities of 52 samples.

**Additional file 5: Table S5.** Positive selection genes identified by Fst and Δπ in cultivated cannabis accessions (Group 1 and Group 2) by comparison of cultivated cannabis and wild cannabis accessions (Group 3 and Group 4).

**Additional file 6:** **Table S6.** The primers and probes used in this study.

**Additional file 7:** **Fig. S1.** Analysis of the differences in the main phenotypic characteristics between wild cannabisand cultivated cannabis. The data represent the means ± SDs. Significant differences were determined using GraphPad Prism 8 software (* indicates *P* <0.05; ** indicates *P* < 0.01; *** indicates *P* < 0.001; **** indicates *P*< 0.0001).

**Additional file 8:** **Fig. S2.** Summary of nucleotide diversity and correlations between latitude and diversity among cultivated cannabis accessions. **A** Nucleotide diversity calculated for each individual and plotted based on different groups interms of population structure. The boxes and inside lines represent quartile ranges and median values, respectively. **B** Scatterplot and linear fitting curve of the latitude and diversity of 13 cultivated cannabis varieties (C1-C6, C9-C12, YNN, GXI and SCN), with some admixed samples removed. **C** Correlations between latitude and diversity among the 13 cultivated cannabis accessions.

**Additional file 9:** **Fig. S3.** Expression of *FT-like* in wild (W4) and cultivated (C4) cannabis accessions grown underLD conditions at different time points on the same day. Thephotoperiod was set such that it was 18 h of light/6 h of darkness (6:00-24:00for light). Samples were taken every three hours. The sampling location was thefirst to second pair of true leaves, from the top down.

## Data Availability

All the data generated or analysed during this study are included in the manuscript and its additional files. The clean sequencing data have been uploaded to the National Genomics Data Center (https://ngdc.cncb.ac.cn/gsa/) under the BioProject ID PRJCA007391. The datasets are available from the corresponding author on reasonable request.

## Abbreviations

THC: Tetrahydrocannabinol
CBD: Cannabidiol
SSR: Simple sequence repeat
SNP: Single-nucleotide polymorphism
N China: Northern China
E China: Eastern China
NW China: Northwestern China
NE China: Northeastern China
SW China: Southwestern China
LD: Long day
SD: Short day
PCA: Principal component analysis
NJ tree: Neighbour-joining tree
qRT–PCR: Quantitative reverse-transcription PCR
PSGs: Positive selection genes
EF1α: Elongation factor 1-alpha
FT-like: Flowering locus T-like
SOC1: Suppressor of overexpression of CO1
FLC-like: Flowering locus C-like
CET1: CEN-like protein 1
PRR37: Two-component response regulator-like PRR37
NFYC9: Nuclear transcription factor Y subunit C-9
FY: Flowering time control protein FY
