# Supplementary material for: Whole-genome resequencing of wild and cultivated cannabis reveals the genetic structure and adaptive selection of important traits
Source: BMC Plant Biol. 2022 Jul 27;22:371. doi: 10.1186/s12870-022-03744-0 (PMC9327241; doi:10.1186/s12870-022-03744-0)
Supplement: Supplementary file 7 — Additional file 7: Fig. S1. Analysis of the differences in the main phenotypic characteristics between wild cannabisand cultivated cannabis. The data represent the means ± SDs. Significant differences were determined using GraphPad Prism 8 software (* indicates P <0.05; ** indicates P < 0.01; *** indicates P < 0.001; **** indicates P< 0.0001). [file 12870_2022_3744_MOESM7_ESM.doc]

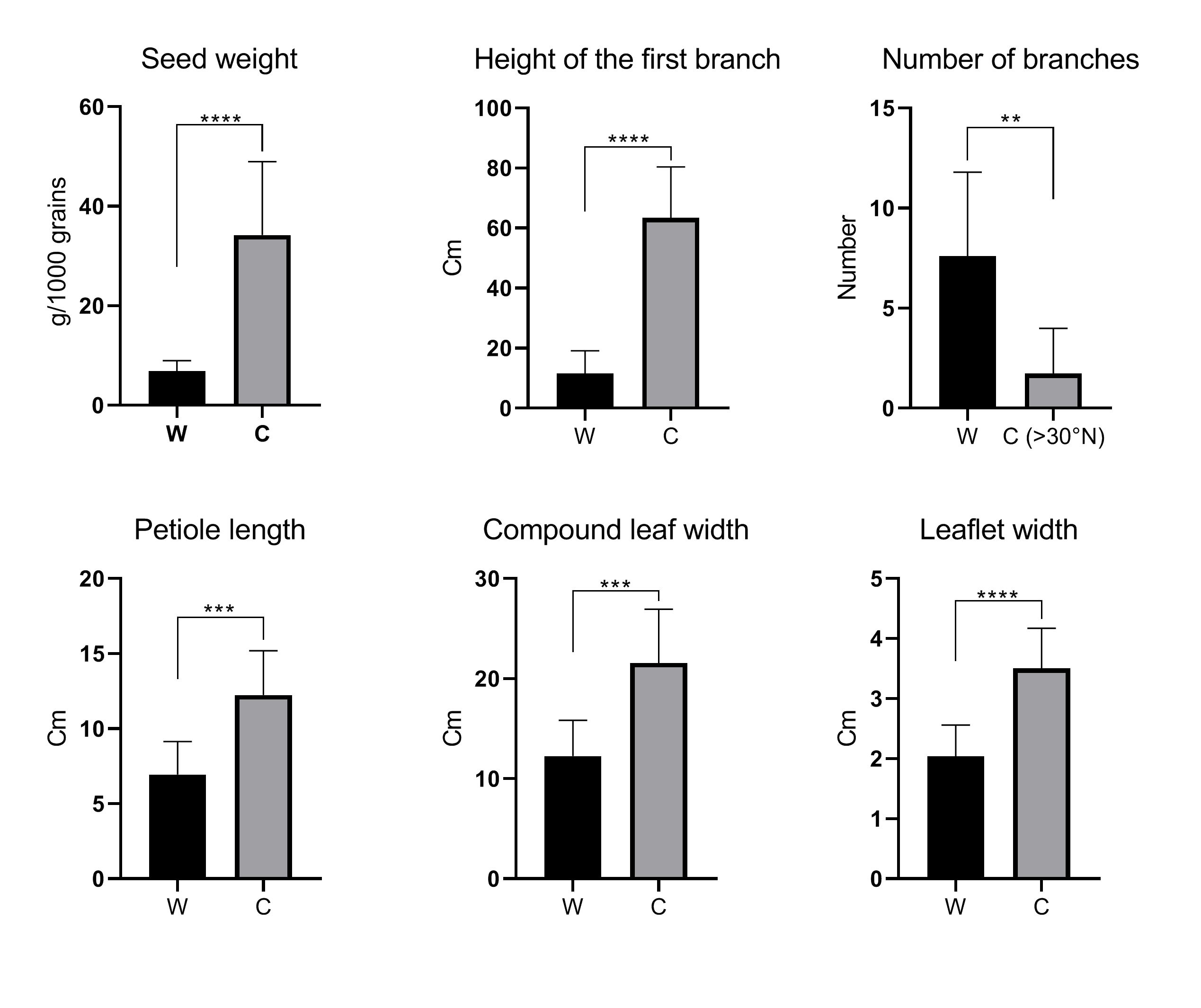


**Fig. S1 Analysis of the differences in the main phenotypic characteristics between wild cannabis and cultivated cannabis.** The data represent the means ± SDs. Significant differences were determined using GraphPad Prism 8 software (* indicates P < 0.05; ** indicates P < 0.01; *** indicates P < 0.001; **** indicates P < 0.0001).
