# Supplementary material for: Whole-genome resequencing of wild and cultivated cannabis reveals the genetic structure and adaptive selection of important traits
Source: BMC Plant Biol. 2022 Jul 27;22:371. doi: 10.1186/s12870-022-03744-0 (PMC9327241; doi:10.1186/s12870-022-03744-0)
Supplement: Supplementary file 8 — Additional file 8: Fig. S2. Summary of nucleotide diversity and correlations between latitude and diversity among cultivated cannabis accessions. A Nucleotide diversity calculated for each individual and plotted based on different groups interms of population structure. The boxes and inside lines represent quartile ranges and median values, respectively. B Scatterplot and linear fitting curve of the latitude and diversity of 13 cultivated cannabis varieties (C1-C6, C9-C12, YNN, GXI and SCN), with some admixed samples removed. C Correlations between latitude and diversity among the 13 cultivated cannabis accessions. [file 12870_2022_3744_MOESM8_ESM.doc]

**
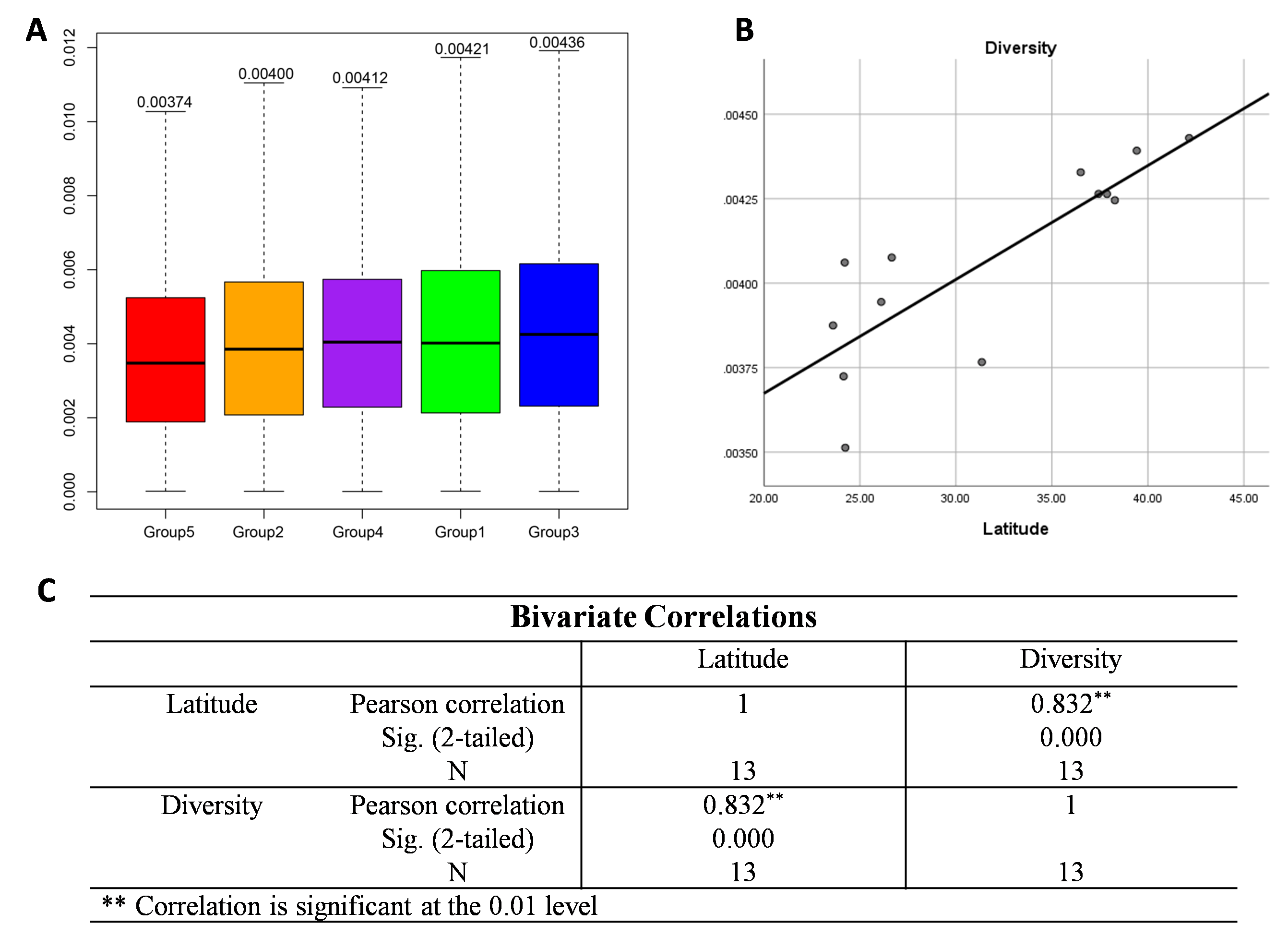
**

**Fig. S2 Summary of nucleotide diversity and correlations between latitude and diversity among cultivated cannabis accessions. A** Nucleotide diversity calculated for each individual and plotted based on different groups in terms of population structure. The boxes and inside lines represent quartile ranges and median values, respectively. **B** Scatterplot and linear fitting curve of the latitude and diversity of 13 cultivated cannabis varieties (C1-C6, C9-C12, YNN, GXI and SCN), with some admixed samples removed. **C** Correlations between latitude and diversity among the 13 cultivated cannabis accessions.
