# Supplementary material for: Whole-genome resequencing of wild and cultivated cannabis reveals the genetic structure and adaptive selection of important traits
Source: BMC Plant Biol. 2022 Jul 27;22:371. doi: 10.1186/s12870-022-03744-0 (PMC9327241; doi:10.1186/s12870-022-03744-0)
Supplement: Supplementary file 9 — Additional file 9: Fig. S3. Expression of FT-like in wild (W4) and cultivated (C4) cannabis accessions grown underLD conditions at different time points on the same day. Thephotoperiod was set such that it was 18 h of light/6 h of darkness (6:00-24:00for light). Samples were taken every three hours. The sampling location was thefirst to second pair of true leaves, from the top down. [file 12870_2022_3744_MOESM9_ESM.doc]

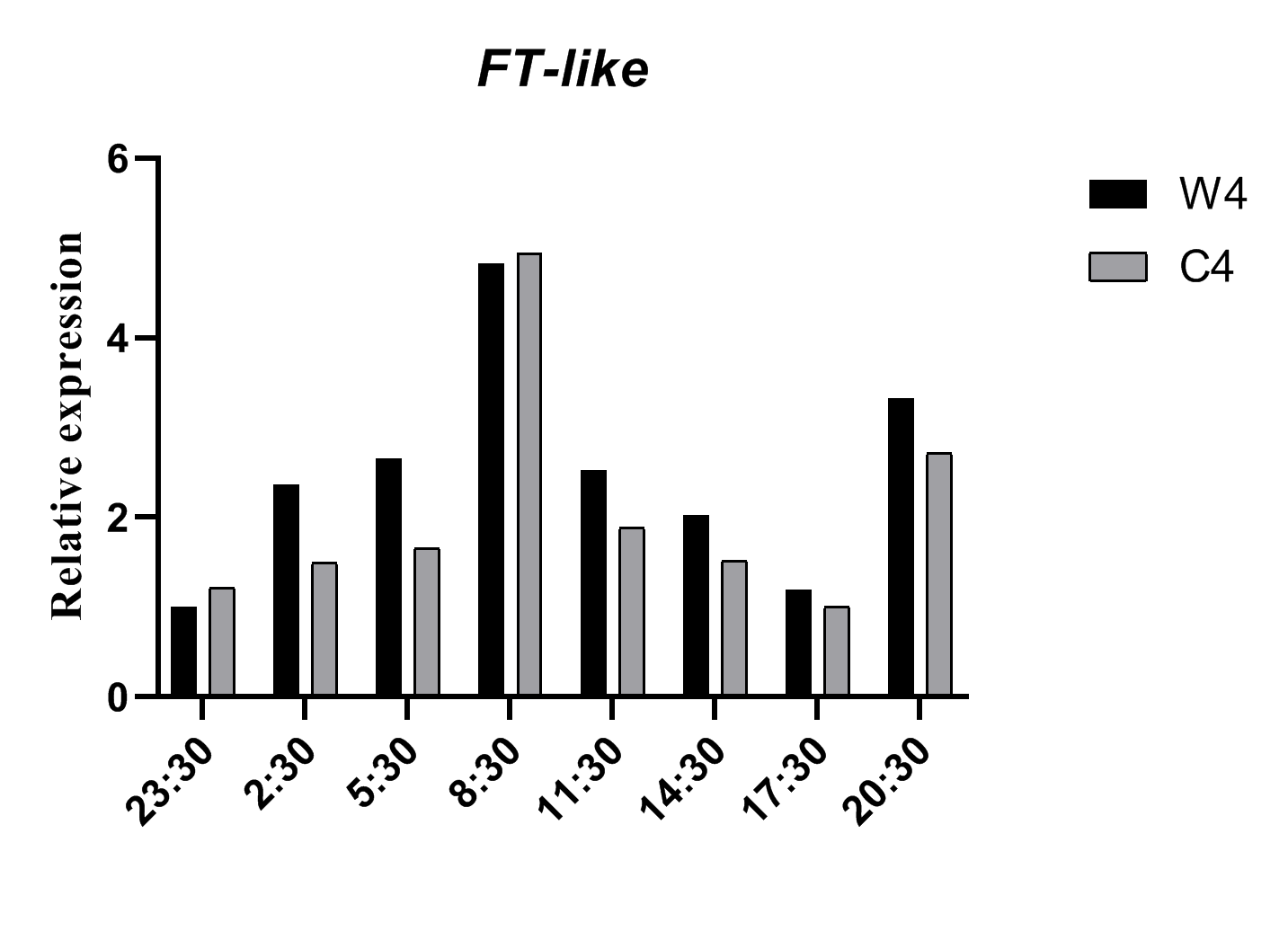


**Fig. S3 Expression of *FT-like* in wild (W4) and cultivated (C4) cannabis accessions grown under LD conditions at different time points on the same day.** The photoperiod was set such that it was 18 h of light/6 h of darkness (6:00-24:00 for light). Samples were taken every three hours. The sampling location was the first to second pair of true leaves, from the top down.
